# Enzymatic Activity of CD73 Modulates Invasion of Gliomas via Epithelial–Mesenchymal Transition-Like Reprogramming

**DOI:** 10.3390/ph13110378

**Published:** 2020-11-11

**Authors:** Julia Tsiampali, Silke Neumann, Beatriz Giesen, Katharina Koch, Donata Maciaczyk, Christoph Janiak, Daniel Hänggi, Jaroslaw Maciaczyk

**Affiliations:** 1 Neurosurgery Department, University Hospital Duesseldorf, 40225 Duesseldorf, Germany; julia.tsiampali@gmail.com (J.T.); Katharina.Koch@iuf-duesseldorf.de (K.K.); daniel.haenggi@med.uni-duesseldorf.de (D.H.); 2 Department of Pathology, University of Otago, Dunedin 9016, New Zealand; silke.neumann@otago.ac.nz (S.N.); donata.maciaczyk@otago.ac.nz (D.M.); 3 Institute of Inorganic Chemistry and Structural Chemistry, Heinrich Heine University Duesseldorf, 40225 Duesseldorf, Germany; beatriz.Giesen@uni-duesseldorf.de (B.G.); janiak@uni-duesseldorf.de (C.J.); 4 IUF-Leibniz Research Institute for Environmental Medicine, 40225 Duesseldorf, Germany; 5 Department of Neurosurgery, University Hospital Bonn, 53179 Bonn, Germany; 6 Department of Surgical Sciences, University of Otago, Dunedin 9016, New Zealand

**Keywords:** A_3_AR, adenosine, CD73, drug targets, epithelial–mesenchymal transition, glioblastoma

## Abstract

Glioblastoma (GBM) is the most aggressive malignant primary brain tumour in adulthood. Despite strong research efforts current treatment options have a limited impact on glioma stem-like cells (GSCs) which contribute to GBM formation, progression and chemoresistance. Invasive growth of GSCs is in part associated with epithelial–mesenchymal-like transition (EMT), a mechanism associated with CD73 in several cancers. Here, we show that CD73 regulates the EMT activator SNAIL1 and further investigate the role of enzymatic and non-enzymatic CD73 activity in GBM progression. Reduction of CD73 protein resulted in significant suppression of GSC viability, proliferation and clonogenicity, whereas CD73 enzymatic activity exhibited negative effects only on GSC invasion involving impaired downstream adenosine (ADO) signalling. Furthermore, application of phosphodiesterase inhibitor pentoxifylline, a potent immunomodulator, effectively inhibited ZEB1 and CD73 expression and significantly decreased viability, clonogenicity, and invasion of GSC in vitro cultures. Given the involvement of adenosine and A_3_ adenosine receptor in GSC invasion, we investigated the effect of the pharmacological inhibition of A_3_AR on GSC maintenance. Direct A_3_AR inhibition promoted apoptotic cell death and impaired the clonogenicity of GSC cultures. Taken together, our data indicate that CD73 is an exciting novel target in GBM therapy. Moreover, pharmacological interference, resulting in disturbed ADO signalling, provides new opportunities to innovate GBM therapy.

## 1. Introduction

Glioblastoma (GBM) is the most common and most aggressive primary brain tumour with a very poor median overall survival of less than two years [1]. Current treatments for GBM include surgical resection of the tumour, followed by radiotherapy and chemotherapy with temozolomide (TMZ) [2]. A subpopulation of cells, glioma stem-like cells (GSCs), characterised by expression of neural stem cell markers and self-renewing capability, contribute to tumour insurgence, progression, recurrence and chemo-resistance [3,4]. Given that GSCs are resistant to the current standard treatment, refined therapeutic approaches targeting this particular subpopulation are needed.

Epithelial-to-mesenchymal-like transition (EMT) has been identified as one of the mechanisms that governs GBM cell dissemination, disease progression and relapse after treatment [5]. During the acquisition of mesenchymal properties, the cells lose their tight connections and become more invasive gaining stem-cell characteristics [6]. EMT-like changes seem to be crucial for tumour initiation, progression, invasion and therapy resistance in GBM [7]. The transcriptional factor Zinc Finger E-Box Binding Homeobox 1 (ZEB1) is one of the main activators of this molecular switch, together with ZEB2, SNAIL1/SNAIL2 and TWIST1 [8,9,10].

Most recently, an ecto-5′-nucleotidase (NT5E) known as CD73 has been shown to regulate EMT, both in ovarian and hepatocellular carcinoma [11,12]. CD73 is a glycosylphosphatidylinositol anchored cell surface protein, which plays crucial roles in the regulation of adenosynergic signalling [13,14]. CD73 possesses an enzymatic and non-enzymatic activity [15,16]. As an enzyme, CD73 catalyses the conversion of adenosine mono phosphate (AMP) to adenosine (ADO) (Figure S1), which is involved in tumour immune escape [17]. AMP can be produced by the conversion of ATP via the ectonucleoside triphosphate diphosphohydrolase 1 (CD39) [18]. In addition to its enzymatic activity, CD73 acts as an adhesive molecule by regulating cell interaction with extracellular matrix components [19,20]. ADO is also a ubiquitous neuromodulator and upstream regulator of diverse brain functions [21]. It signals through four adenosine receptors (ARs): A_1_, A_2_A, A_2_B, and A_3_ [22]. Within the tumour microenvironment, high ADO concentrations activate its low affinity receptors A_3_ and A_2B_ [23]. In GBM, extracellular ADO has been shown to promote the invasive capacity via A_3_AR signalling and modulate EMT-like processes via both the A_3_AR the A_2B_AR [24,25]. Therefore, CD73 has emerged as an interesting target in the treatment of GBM.

However, the role of the enzymatic and non-enzymatic function of CD73 in GBM progression has not been fully elucidated. Furthermore, it still remains to be clarified how CD73 activity affects the maintenance of highly aggressive and chemo-resistant GSCs. Therefore, we investigated the effect of CD73 modulation on GSCs growth and highlighted its potential as a novel therapeutic target for glioma therapy.

## 2. Results

### 2.1. CD73 Is Expressed in Hypoxic Areas of GBMs and Affects EMT-Like Reprogramming

**Figure 1:**
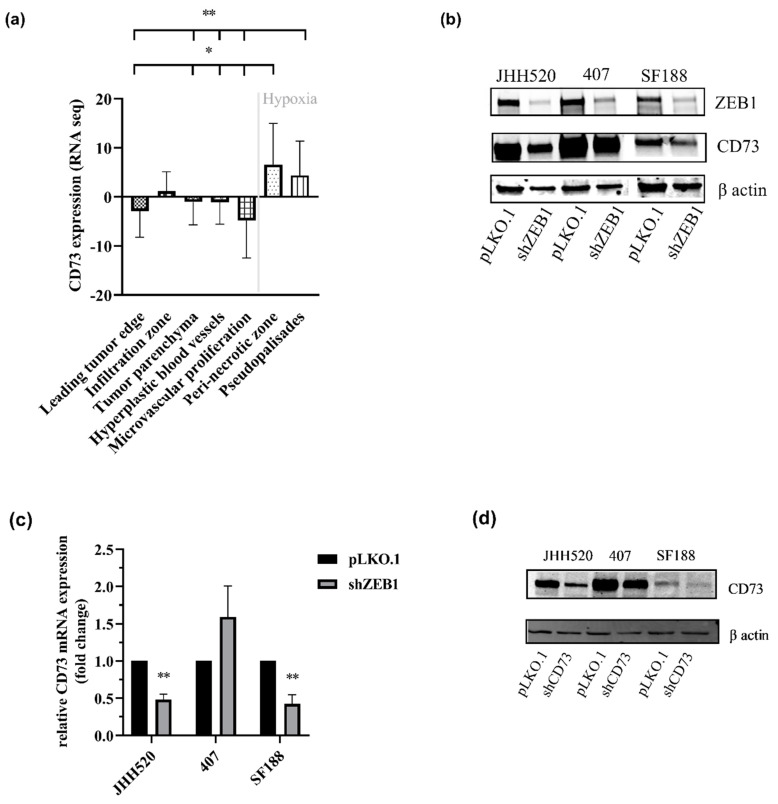
CD73 increases in hypoxia and is regulated by epithelial-to-mesenchymal-like transition (EMT). (**a**) mRNA expression data were retrieved from the IVY Glioblastoma project and analysed for CD73 expression (*n* = 55 samples). CD73 mRNA was increased in the hypoxic pseudopalisades and peri-necrotic areas of glioblastoma (GBM) samples. Statistical significance was calculated with one-way ANOVA. (**b**) CD73 protein levels upon Zinc Finger E-Box Binding Homeobox 1 (ZEB1) knockdown were analysed in glioma stem-like cell (GSC) lines JHH520, 407 and SF188 by Western blotting (β-actin, loading control) (**c**). CD73 mRNA levels were analysed in three GSC lines and found to be decreased in two cell lines (JHH520 and SF188, *p* < 0.001) upon inhibition of ZEB1. (**d**) The CD73 knockdown efficiency in GSC lines (JHH520, 407, SF188) was confirmed by Western blotting. Statistical significance was calculated with unpaired t-tests. Results are presented as mean ± SD of three independent experiments performed in triplicate. * *p* < 0.05, ** *p* < 0.001.

When investigating the location of CD73 expression, we found that CD73 was predominantly expressed in hypoxic regions of the brain (Figure 1a) such as the pseudopalisades and perinecrotic zone that contain high numbers of invasive cells [26]. Since ZEB1 is a pivotal player in EMT in these regions [27], we evaluated the effect of ZEB1 knockdown on CD73 expression. In addition, most recently, CD73 has been shown to regulate EMT, both in ovarian and hepatocellular carcinoma [11,12]. We found that ZEB1 knockdown was associated with a reduction of CD73 expression on the protein (Figure 1b) and mRNA level (Figure 1c).

### 2.2. CD73 Inhibition Reduces the Viability, Clonogenic and Invasive Capacities of GBMs

**Figure 2:**
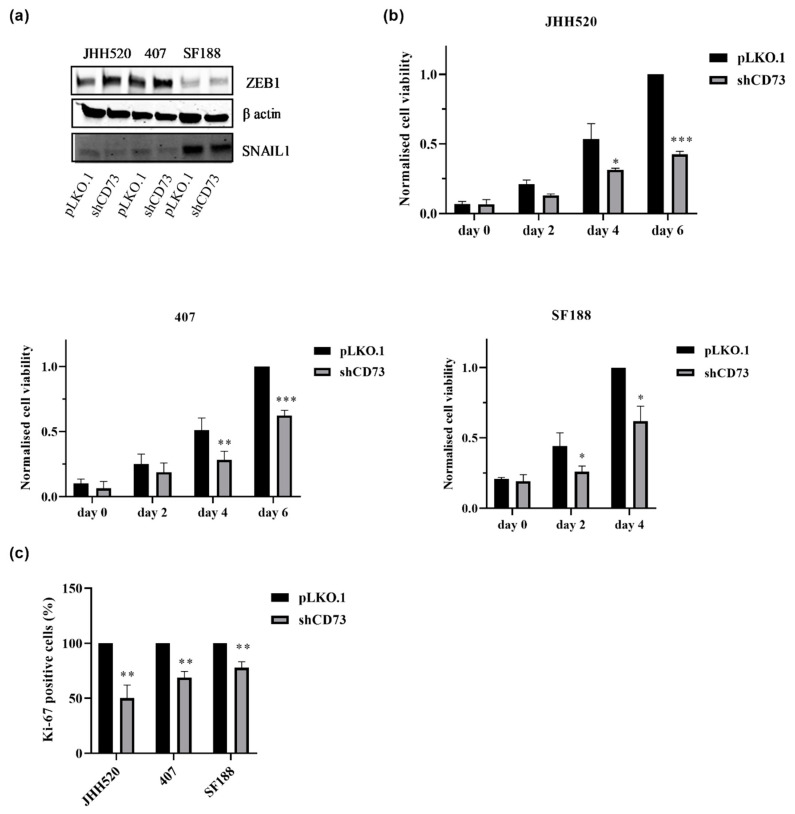
CD73 inhibition reduces the viability of GSC cultures and regulates EMT via SNAIL1 protein suppression. (**a**) SNAIL1 but not ZEB1 was affected by CD73 depletion as assessed by Western blotting upon CD73 knockdown. (**b**) The cell viability and (**c**) proliferation in CD73 knockdown cells were reduced as compared to control cells (pLKO.1). Statistical significance was calculated with unpaired t-tests. The results are presented as mean ± SD of three independent experiments performed in triplicate. * *p* < 0.05, ** *p* < 0.005, *** *p* < 0.0001.

**Figure 3:**
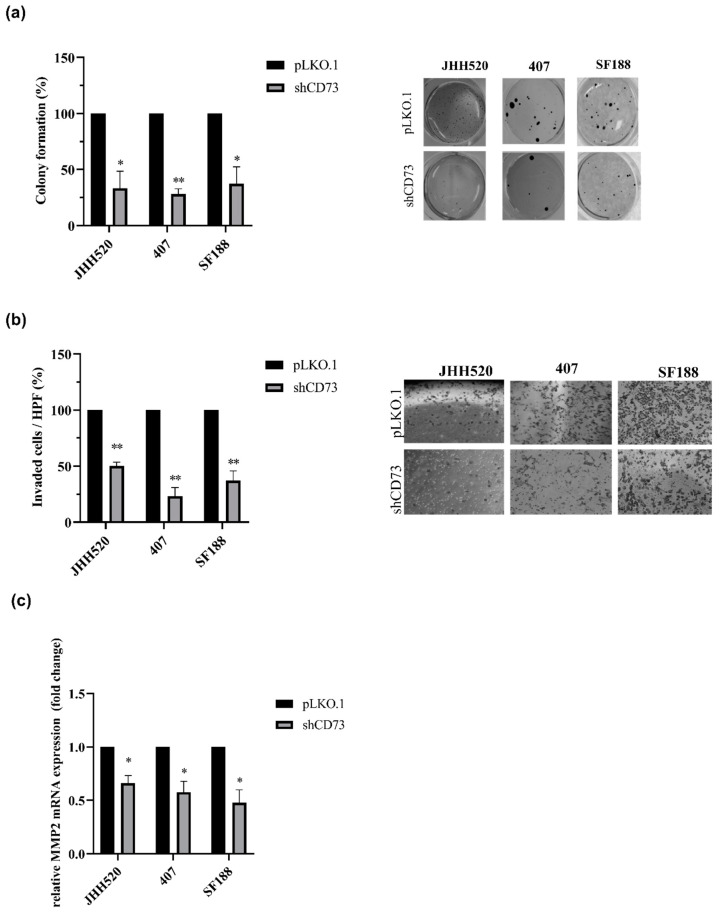
CD73 inhibition reduces clonogenicity and invasion of GSC cultures. (**a**) Knockdown of CD73 reduced colony formation of JHH520, 407 and SF188 cells as assessed by soft agar assays. (**b**) CD73 suppression decreased the number of invasive cells after 24 h (**c**) and MMP2 mRNA expression levels compared to control (pLKO.1). Abbreviations: HPF, high power field. Statistical significance was calculated with unpaired t-tests. The results are presented as mean ± SD of three independent experiments performed in triplicate. * *p* < 0.005, ** *p* < 0.001.

In order to analyse whether CD73 is involved in the maintenance of chemo-resistant GSCs, we established GSC-enriched in vitro cultures with suppressed expression of CD73 (JHH520, 407 and SF188) using RNA interference. The shRNA efficiency was validated on the mRNA (data not shown) and protein level (Figure 1d). When testing the effect of CD73 suppression on EMT activators, we found that CD73 suppression decreased the protein expression of SNAIL1, an EMT regulator but not ZEB1 (Figure 2a). To investigate the biological effect of CD73 reduction, cell viability of CD73 knockdown and control cells was assessed. CD73 knockdown decreased the viability of all three tested GSC lines (Figure 2b, *p* < 0.05). Furthermore, we tested the effect of the CD73 knockdown on the proliferation of all three cell lines by Ki-67 staining, which is a marker of cell cycle progression [28]. Ki‑67 was decreased in CD73 knockdown cells indicating a reduced proliferation rate (Figure 2c, *p* < 0.002). Similarly, the clonogenic capacity of GSCs was reduced by more than 50% upon CD73 knockdown (Figure 3a). Moreover, we tested the invasive properties after CD73 depletion using a modified Boyden chamber assay. We found that CD73 knockdown decreased the number of invading cells by at least 50% in all three cell lines tested (Figure 3b). In addition, analysis of matrix metalloproteinase-2 (MMP-2, an enzyme involved in tumour invasion) mRNA expression showed significantly decreased MMP-2 mRNA transcription upon CD73 inhibition (Figure 3c, *p* < 0.001).

### 2.3. The Enzymatic Activity of CD73 Promotes the Invasive Properties of GSCs

**Figure 4:**
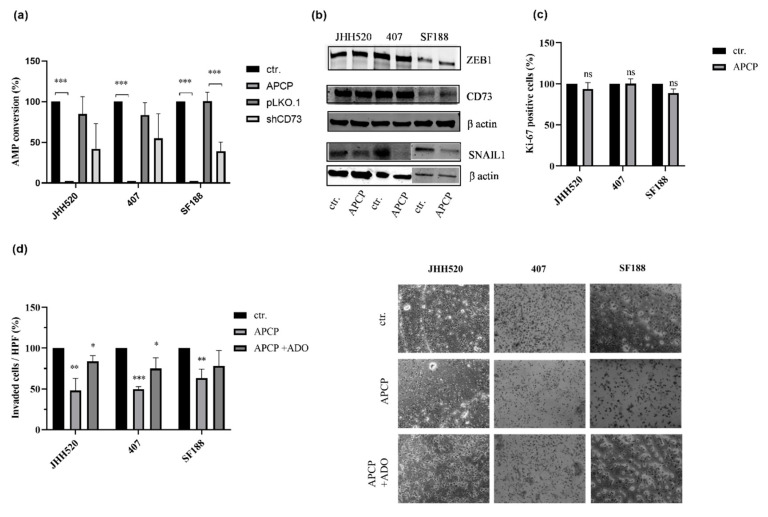
CD73 enzymatic activity does not affect GSC proliferation but reduces their invasive properties. (**a**) The percentage (%) of AMP conversion was measured in empty vector control (pLKO.1) cells, in CD73 knockdown (shCD73) cells, in wild type control (ctr.) cells and 10 µM APCP-treated (APCP) control cells. (**b**) ZEB1 and SNAIL1 protein expression levels were determined using immunoblotting in APCP (10 μM) for 24 h and untreated control cells (ctr.) (β-actin, loading control). Representative blots are shown. (**c**) The percentage of the Ki-67-positive cells upon treatment with APCP was not significantly decreased compared with untreated control cells (ctr.). (**d**) Inhibition of CD73 enzymatic activity with APCP significantly decreased the number of invaded cells after 24 h when compared with untreated cells (ctr.). Addition of 10 µM adenosine (ADO) restored the invasive capacity of APCP-treated cells. Abbreviations: HPF, high power field; ns, not significant. Statistical significance was calculated with unpaired t-tests. Results are presented as mean ± SD of three independent experiments performed in triplicate. * *p* < 0.05, ** *p* < 0.005, *** *p* < 0.001.

To investigate whether the enzymatic or non-enzymatic activity of CD73 is responsible for maintaining stem-cell characteristics of GSC cultures, we measured changes in the conversion of AMP to ADO upon genetic and pharmacological inhibition of CD73 using HPLC. The separation method used was able to detect both compounds in the same sample (Figure S2a) and quantification was possible through calibration of AMP and ADO standards. To illustrate the HPLC separation of cell supernatants, an exemplary chromatogram of JHH520 cells after incubation with AMP is shown in Figure S2b. Reduction of CD73 protein expression led to a decrease in AMP conversion by the cells (Figure 4a, *p* < 0.001 for SF188). The pharmacological inhibitor of CD73 enzymatic activity, APCP (10 µM), completely blocked the conversion of AMP to ADO (Figure 4a, *p* < 0.0001). In concordance with the CD73 knockdown, it reduced expression of the EMT factor SNAIL1 (Figure 4b).

Aiming to determine the role of CD73 enzymatic activity in glioma progression, all three GSC lines were treated with increasing concentrations of the APCP (1–50 μM). The cell viability was assessed after a duration of 2, 4 and 6 days of treatment. Inhibition of CD73 enzymatic activity did not affect the survival of GSCs (Figure S3a). Similarly, inhibition of the enzymatic activity did not reduce the proliferation of GBMs (Figure 4c). Moreover, treatment with APCP did not change the clonogenic properties of the tested cell lines 407 and SF188 and it even slightly increased the clonogenic properties of JHH520 by 11. 6% (Figure S3b). In contrast, the enzymatic activity of CD73 was crucial for the invasive properties of GSCs (Figure 4d). Furthermore, addition of extracellular ADO effectively restored the reduced invasive capacity of glioma cells upon APCP treatment (Figure 4d). Taken together, our results indicate that CD73 increases the invasiveness of GBMs via its enzymatic activity and downstream ADO signalling.

### 2.4. The Phosphodiesterase Inhibitor Pentoxifylline Supresses ZEB1 and CD73 Expression in GSC Cultures

**Figure 5:**
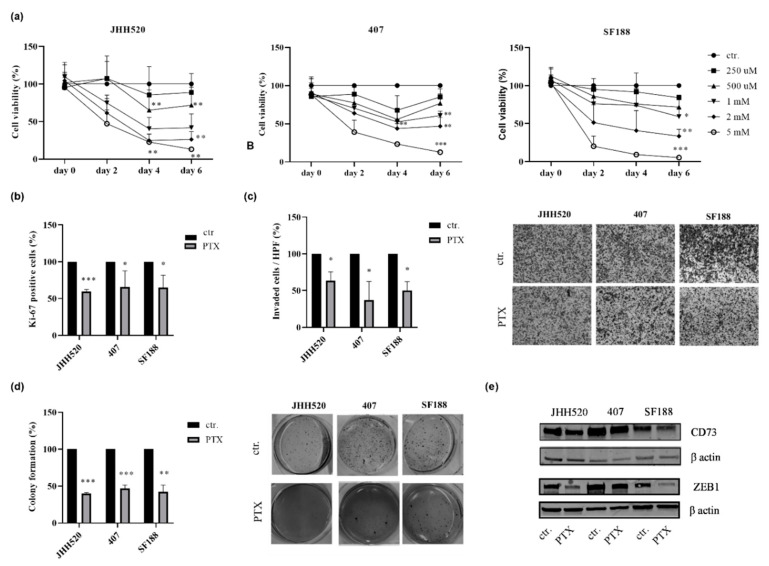
Pentoxifylline (PTX) inhibits ZEB1 and CD73 expression and reduces the viability of GSCs. (**a**) GSC lines (JHH520, 407 and SF188) were treated with increasing concentrations of PTX (250 μM - 5 mM) and the percentage (%) of cell viability was assessed compared to control (ctr.). (**b**) The percentage of Ki-67-positive cells was reduced in the PTX-treated cells (JHH520 1 mM, 407 and SF188 2 mM) compared to untreated cells (ctr.). (**c**) The invasive capacity of GSCs was also significantly decreased upon PTX treatment (JHH520 1 mM, 407 and SF188 2 mM) cells (ctr.). (**d**) The clonogenic capacity of cells was assessed by soft agar assays. Treatment with PTX led to a lesser degree of colony formation (JHH520 1 mM, 407 and SF188 2 mM) as compared to untreated cells (ctr.) (**e**) Lower ZEB1 and CD73 protein expression levels were detected using immunoblotting in PTX-treated cells compared to the control cells (ctr.). Abbreviations: HPF, high power field. Statistical significance was calculated with unpaired t-tests. Results are depicted as mean ± SD of three independent experiments performed in triplicate. * *p* < 0.005, ** *p* < 0.001, *** *p* < 0.0001.

The clinically approved compound pentoxifylline (PTX), which is clinically used as vasodilator to increase blood flow and tissue oxygenation [29], has been reported to inhibit dephosphorylation of AMP to ADO via inhibition of 5′-nucleotidase [30]. In addition, due to its properties as a phosphodiesterase inhibitor, PTX positively regulated the intracellular levels of the second messenger cyclic adenosine monophosphate (cAMP) that can affect the EMT mechanism [31,32]. Therefore, we were interested in assessing the effect of this drug on the growth of investigated GSC cultures and ZEB1 protein expression. Treatment with PTX (1 mM for JHH520 and 2 mM for 407 and SF188) decreased the viability of GBMs (Figure 5a) and reduced their proliferation (Figure 5b). Furthermore, the clonogenic and invasive capacities of GSCs were significantly decreased following PTX treatment (Figure 5c,d, *p* < 0.0001). Treatment with PTX (1 mM for JHH520 and 2 mM for 407 and SF188) inhibited ZEB1 and CD73 expression (Figure 5e). These data support the use of PTX for targeting GBMs.

### 2.5. Pharmacological Inhibition of the A_3_A Receptor Effectively Targets GSCs

**Figure 6:**
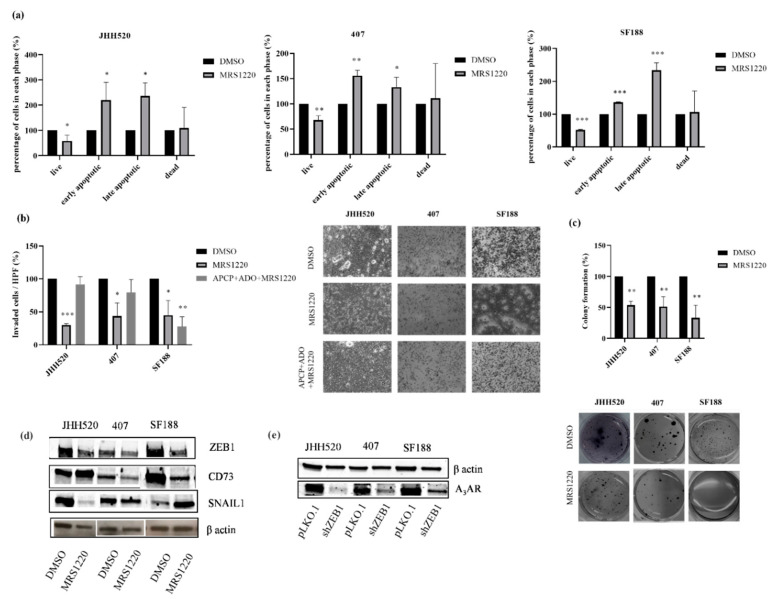
Pharmacological inhibition of the A_3_A receptor reduces stemness and mesenchymal characteristics in GSCs. (**a**) Pharmacological inhibition of A_3_AR with 10 μM MRS1220 for 24 h induced apoptosis as assessed with the Muse^®^ Annexin V and Dead Cell Kit. Data normalised over the DMSO controls. (**b**,**c**) MRS1220 (10 μM) reduced the invasive and clonogenic capacities of GSCs. (**d**) ZEB1 protein expression levels were decreased in MRS1220 (10 μM)-treated cells comparing to the control (DMSO) whereas CD73 and SNAIL1 protein expression levels showed no common features. (**e**) ZEB1 inhibition decreased the protein expression of A_3_AR. Abbreviations: HPF, high power field. Statistical significance was calculated with unpaired t-tests. The results are depicted as mean ± SD of three independent experiments performed in triplicate. * *p* < 0.05, ** *p* < 0.005, *** *p* < 0.0001.

ADO, the product of CD73 enzymatic activity, has been shown to promote cell invasion in GSCs via A_3_AR [24]. To evaluate the role of A_3_AR in GBM growth and EMT modulation, we treated the GSC cultures with the A_3_AR antagonist MRS1220. Treatment with increasing concentrations of MRS1220 led to decreased survival (Figure S4, *p* < 0.001). It was further associated with apoptosis as assessed by an increase in cell numbers staining positive for Annexin V (+) and 7-AAD (−) indicating early apoptotic cells and the late apoptotic cells Annexin V (+) and 7-AAD (+) as compared to untreated cells (Figure 6a, *p* < 0.05). Furthermore, treatment with MRS1220 decreased the clonogenic and invasive capacities of the glioma cells (Figure 6b,c, *p* < 0.005 and *p* < 0.05, respectively). Next, we investigated whether the A_3_AR could be the main ADO receptor responsible for GBMs invasive capacities. Therefore, GSCs were treated with the A_3_AR antagonist MRS1220 with or without the addition of the inhibitor of CD73 enzymatic activity APCP and ADO. We found that the A_3_AR antagonist MRS1220 did not decrease the number of invading cells at the presence of 10 µM APCP and 10 µM ADO when compared with vehicle treated cells (DMSO). However, in paediatric GBM cell line SF188, adding MRS1220 to APCP and ADO significantly decreased the invasion of the cells (Figure 6b, *p* < 0.005).

To investigate the effect of A_3_AR blockade on EMT, we tested the protein expression of EMT markers after treatment with the A_3_AR inhibitor MRS1220. We found that blockade of A_3_AR reduced ZEB1 expression (Figure 6d). Interestingly, blockade of A_3_AR decreased both CD73 and SNAIL1 protein expressions in 407. However, the effect of A_3_AR blockade on CD73 and SNAIL1 was variable and no common features could be found in the tested cultures (Figure 6d). Furthermore, EMT modulation led to a decrease in A_3_AR protein expression (Figure 6e).

## 3. Discussion

In this study, we highlight the role of both the enzymatic and non-enzymatic activity of CD73 on the maintenance of highly chemo-resistant GSCs, that are positive for the stemness markers CD133 and SOX2 [33]. Decreased CD73 expression caused a significant suppression of GSC clonogenicity, cell invasion and proliferation [15,34]. More specifically, we showed that the effects on GSC proliferation and clonogenicity were independent from its enzymatic activity but dependent on the CD73 protein level. However, in contrast to previous findings [34,35], selective inhibition of CD73 enzymatic activity by APCP had significant effects only on the invasiveness of GSCs. In concordance, addition of the CD73 product ADO efficiently rescued the APCP phenotype. These results indicate that the CD73 enzymatic activity is a possible mechanism contributing significantly to the invasiveness of GBMs. In GBM and breast cancer, CD73 regulates tumour invasion via matrix metalloproteinases (MMPs) by degrading the extracellular matrix (ECM) [19,34]. Our results also showed decreased MMP2 mRNA expression upon CD73 knockdown, indicating the regulation of invasion via this metalloproteinase, as one of the aspects of the possible mechanism. In hepatocellular carcinoma, CD73 promoted invasion of the cells via activating PI3K/AKT signalling [12]. Interestingly, it has also been reported that CD73 promotes invasion and metastasis of head and neck squamous cell carcinoma (HNSCC) by stimulating the adenosine A_3_ receptor [36].

We showed, that CD73 protein expression was reduced upon inhibition of the EMT activator ZEB1. Interestingly, both CD73 knockdown and suppression of the CD73 enzymatic activity with APCP reduced the protein expression of the EMT regulator SNAIL1 in all three GSC lines. Similarly, knockdown of CD73 in HNSCC has been suggested to regulate EMT via SNAIL1 and TWIST1 modulation [36]. Our data suggest that there is a reciprocal interaction between EMT-like processes and CD73 enzymatic activity in GSCs. SNAIL1 has been described to promote the invasive and clonogenic capacities of GBM tumours [37]. Thus, the reduced SNAIL1 expression diminishes mesenchymal properties of CD73 knockdown cells, demonstrated by their significantly decreased invasiveness and clonogenicity. Previous studies of our laboratory identified further metabolic enzymes directly correlating EMT-like processes with stemness and chemo-resistance of GSC cultures, which indicates that the metabolic homeostasis is pivotal for the maintenance of GSCs and the metabolic enzymes may act as promising targets in GBM therapy [33,38].

Since the enzymatic activity of CD73 and CD39 drives the production of immunosuppressive ADO, CD73 and CD39 have been identified as a novel immune checkpoint targets [17,39,40]. Both CD73 and CD39 can be also expressed on immune cells [41]. Furthermore, CD73 and CD39 play an important role in the ramification of microglia process through the ADO generated in the microglia microenvironment [42]. The clinical approved drug PTX reduced the survival of GSC cultures and the protein expression of ZEB1 and CD73 protein. PTX increases the intracellular levels of cAMP and as a result it affects EMT [32]. Therefore, our results showed that PTX reduced ZEB1 and as a consequence protein levels of CD73 were also decreased. As expected, treatment with PTX showed a decrease in GSC growth, invasiveness and clonogenicity. It has previously been shown that PTX abolished the radiation-induced G2/M block in GBMs and reduced β-catenin activity in melanoma cells [29,43]. Interestingly, PTX possess anti-inflammatory properties that are mostly associated with the downregulation of TNF-α synthesis [31]. Taken together, PTX has been identified as a potentially effective compound against GBM. Here we show that its efficacy may at least partially be mediated via EMT activator ZEB1 and downstream CD73.

The A_3_AR is an interesting receptor in GBM since it has been shown to promote tumour invasiveness and to increase multiple drug resistance protein-1 (MRP1) expression and GBM proliferation following chemotherapeutic treatment [24,44]. Indeed, our results indicate the involvement of A_3_AR in regulating invasion and clonogenicity of GSCs. Therefore, ADO signalling is a possible mechanism contributing significantly to the promotion of mesenchymal properties of cells. Interestingly, we observed that blockade of the A_3_AR in the paediatric GSC line SF188 constantly inhibited the invasive properties of this cell line, whereas the effect in adult-derived JHH520 and 407 cells could be rescued by supplementation with ADO. Importantly, A_3_AR inhibitor MRS1220 is an orthosteric inhibitor; therefore, given its properties, there are no allosteric changes at the binding site of the receptor and ADO can rescue the effect [45]. This could indicate the importance of further ADO receptors on these cell lines playing a role in promoting invasion and underpinning the possible differences between paediatric and adult gliomas.

We also showed that A_3_AR is important in regulating EMT via ZEB1. The A_3_AR antagonist MRS1220 reduced the protein expression of the EMT activator SNAIL1 in cell lines SF188 and 407 and decreased expression of CD73 in JHH520 cells. In addition, it had been shown that A_3_AR blockade in GBM decreased the expression of SNAIL1 under hypoxia [24]. These results indicate that A_3_AR regulates EMT globally by affecting the expression of several EMT activators also independent of CD73. Interestingly, treatment of GBM cells with the A_3_AR antagonist significantly decreased the viability of the cells by inducing apoptosis. The effect of A_3_AR on cell proliferation and apoptosis had been reported to be both positive and negative depending on several factors such as agonist concentration, cell type and tumour microenvironment [46]. In prostate cancer cells, A_3_AR activation inhibited PKA-mediated ERK 1/2 activation and subsequent NADPH oxidase activities, resulting in decreased proliferation and invasion of cells [47]. In another study, activation of A_3_AR using a selective agonist led to decreased proliferation of melanoma cells through inhibition of the phosphorylation or inactivation of GSK-3β that induced the phosphorylation or inactivation of β-catenin [48]. The greatest challenge is still to understand the conditions in which selective A_3_ agonists or antagonists would be of benefit [49]. In view of the data presented here, A_3_AR could be an interesting target in GBM therapy.

One of the biggest challenges in GBM therapy is the presence of the blood–brain barrier (BBB) and in addition to that, it has been shown that small molecules can be removed from the brain in a very short period even after stereotactic delivery [50]. These limitations need to be overcome for the clinical setting of targeting CD73 and A_3_AR. However, antibodies that are larger molecules could be more effective as shown in murine tumour models [51]. Recent data suggest that the retention time in the brain was increased upon effective delivery of radiolabelled antibodies [52].

The exact mechanism of how CD73 suppresses the EMT activator SNAIL1 and decreases the GSC cell growth, clonogenic and invasive capacities still needs to be elucidated. In human breast cancer, CD73 had a regulatory effect on EGFR expression and phosphorylation, which correlated with tumour growth [53]. It has been shown that CD73 expression was higher in more malignant (higher expression of mesenchymal markers) breast cancer cells and its expression increased significantly in TGF-β-induced EMT cells [54]. The influence of CD73 on EMT could be studied in association with TGF-β, the most common EMT-inducing factor [55,56]. In colorectal cancer, CD73 downregulated cell growth via EGFR and the β-catenin/cyclin D1 signalling pathway [57]. However, in GBM, the enzymatic activity of CD73 and the production of ADO seem to be involved in the regulation of EMT and invasiveness. In conclusion, our data support the potential role of CD73 as a promising target for GBM therapy. Inhibition of CD73 may efficiently lead to suppression of EMT-like processes and eradication of GSCs in malignant gliomas.

## 4. Material and Methods

### 4.1. Cell Culture

Three human glioblastoma cell lines were used in this study. JHH520 cells were generously provided by G. Riggins (Baltimore, MD, USA), 407 (BTSC-407) by M.S. Carro (Freiburg, Germany) and the paediatric GBM cell line SF188 was provided by E. Raabe (Baltimore, MD, USA). The GSC neurospheres were cultured in DMEM without pyruvate (Gibco BRL, Eggenstein, Germany), 30% Ham’s F12 Nutrient Mix (Gibco), 20 ng/mL human recombinant bFGF (Peprotech, Rocky Hill, NJ, USA), 20 ng/mL human recombinant EGF (Peprotech), 5 µg/mL Heparin (Sigma-Aldrich, St. Louis, MO, USA), 2% B27 supplement (Gibco) and 1× Penicillin-Streptomycin (Gibco). HEK293T cells (RRID:CVCL_0063) were purchased from American Tissue Culture Collection (Manassas, VA, USA). HEK293T cells were cultured in DMEM with pyruvate (Gibco) plus 10% Foetal Bovine Serum (FBS; Biochrome, MD, USA) and 1× Penicillin-Streptomycin (Gibco).

All cell lines were routinely tested for the absence of mycoplasma contamination and they were authenticated using short-tandem repeat (STR) profiling.

### 4.2. Generation of Lentiviral Particles

A third-generation lentiviral packaging system was used for the generation of the lentiviral particles as previously described [8]. Briefly, HEK293T cells were transfected with the lentiviral target vector and the three packaging plasmids (pMDLgpRRE, pRSVREV and pMD2VSVG) using FuGENE^®^ HD transfection reagent (Promega, Madison, WI, USA). Supernatants containing the viral particles were collected after 48, 72 and 96 h post transfection and passed through a 0.45-micron filter before being concentrated using polyethylene glycol and sodium chloride (NaCl). Viral particles were stored at −80 °C until needed. The CD73 knockdown was achieved by cloning shRNA into the pLKO.1 TRC vector (Addgene plasmid, Addgene, Cambridge, MA, USA) [58]. Plasmids containing shRNA against ZEB1 were created as described previously [5].

### 4.3. Quantitative Real Time PCR (RT qPCR)

RNA extraction was performed using the RNeasy Mini Kit (Qiagen, Hilden, Germany) following the manufacturer’s instructions. RNA concentrations were measured using the Nanodrop2000 spectrometer (Thermo Scientific, Waltham, MA, USA). Two micrograms of RNA were transcribed into cDNA using the reverse transcriptase M-MLV (Promega) and random hexameric primers. For each experiment, advanced SYBR Green Supermix (BioRad, Hercules, CA, USA), 10 ng of cDNA and 10 pmol of each primer were combined and analysed in a CFX Connect Thermocycler (BioRad). The relative expression levels of genes were normalised to the endogenous housekeeping gene β-actin. Calculation of normalised relative gene expression was performed using the supplied software of the CFX Connect Real-Time PCR Detection System (Bio-Rad) The Primer sequences can be found in Table 1.

### 4.4. Western Blotting

GSCs were lysed in cold RIPA Buffer and the protein concentrations were determined using the DC Protein Assay Kit (BioRad) according to the manufacturer´s instructions. Primary antibodies (CD73, 1/1000, (#ab133582, Abcam, Cambridge, MA, USA); ZEB1, 1/1000 (#HPA027524, Sigma-Aldrich); SNAIL1 1/1000 (#3879, Cell Signalling Technology, Beverly, MA, USA); A_3_AR, 1/1000 (#600445, Biocompare, San Diego, CA, USA); β-actin, 1/1000 (#sc-130657, Santa Cruz Biotechnology, Santa Cruz, CA, USA)) were diluted in blocking solution containing 5% milk powder in Tris-buffered saline with Tween20 (TBST). The membranes were incubated with the respective primary antibodies overnight at 4 °C. Secondary antibodies (goat-anti-rabbit (#926-32211, IRDye800C LI-COR); goat-anti-mouse (#926-68070, IRDye680RD LI-COR), and goat-anti-rabbit-HRP (#111-035-144, Jackson ImmunoResearch, West Grove, PA, USA )) were prepared in blocking solution in a dilution of 1/10,000 and the membranes were incubated with them for 1 h at room temperature. The band signals were acquired by a luminescence-based system in a LI-COR Odyssey CLx Imager (LI-COR) or by film-based system in a Super Signal West Pico Chemiluminescent Substrate (Thermo Scientific). Band quantification was performed using the Image Studio Lite Software version 2.1 (BioRad).

### 4.5. Cell Viability, Proliferation and Cell Death Assays

GSCs were seeded at a density of 2 × 10^4^ cells/mL and treated with inhibitor of CD73 enzymatic activity adenosine 5′-(α,β-methylene)diphosphate (APCP) or phosphodiesterase inhibitor pentoxifylline (PTX) at the following concentrations: 5 μM, 10 μM, 20 μM and 50 μM for APCP and 1 mM for PTX or vehicle (H_2_O). Cells were treated with 1 μM, 5 μM, 10 μM, 20 μM and 50 μM of A_3_AR antagonist MRS1220 or vehicle (DMSO). The viability of the glioma cells was assessed using the Thiazolyl Blue Tetrazolium Bromide assay (MTT, Sigma Aldrich, St Louis, MO, USA) according to the manufacturer’s instructions. The absorbance was measured at 570 nm (reference 650 nm) using a Paradigm™ multiplate reader (Beckman Coulter, Brea, CA, USA).

The percentage of the proliferating cells was determined based on Ki-67 expression using the Muse^®^ Ki-67 Proliferation Kit (Merck Millipore, Burlington, MA, USA) according to the manufacturer’s instructions. Briefly, pLKO.1 and shCD73 cells or cells cultured in medium containing 10 μM APCP or vehicle (H_2_O) were collected. The cells were then fixed and incubated with Ki-67-PE antibody for 30 min at room temperature. Cells were then analysed using the Muse cell analyser (Merck Millipore).

The MUSE Annexin V & Dead Cell Kit (Merck Millipore) was used in order to measure the percentage of apoptotic cells, according to the manufacturer’s instructions. Briefly, cells cultured in medium containing 10 μM MRS1220 or vehicle (DMSO) for 24 h were collected. They were then diluted with PBS containing 1% FBS to a concentration of 2 × 10^5^ cells/mL. An aliquot of 100 μL of this single cell suspension was mixed with 100 μL of Annexin V/dead reagent and kept in the dark for 20 min at room temperature. The analysis of the cells was performed using the Muse cell analyser (Merck Millipore).

### 4.6. Invasion Assay

The invasive capacity of GSCs was evaluated using a modified 24-well Boyden Chamber assay as described previously [38]. The inserts were coated with growth factor-reduced Matrigel (BD, Franklin Lakes, NJ, USA) and were incubated for 1 h at 37 °C. An aliquot of 4 × 10^4^ cells was re-suspended in 500 μL DMEM (Life Technologies, Carlsberg, CA, USA) and placed on top of each insert membrane. The bottoms of the wells were filled with 800 μL DMEM media with 10% FBS. After a 16 h incubation period, the non-invaded cells on the upper surface of the membrane were removed carefully with cotton swabs. The filters of the inserts were fixed in ice-cold methanol for 10 min, washed with PBS and stained with Haematoxylin for 5 min. The invasive capacity of the cells was evaluated by taking 5 pictures per well and counting the stained cells using the ImageJ Program 1.8.0. (Rasband, W.S., U. S. National Institutes of Health, Bethesda, MD, USA). For the drug treatment experiments, cells were pre-treated for 24 h with 10 μM APCP with or without ADO or 10 μM MRS1220 in standard culture conditions before assessing their invasiveness.

### 4.7. Clonogenicity Assay

The clonogenic capacity of glioma cells was measured by performing soft agar assays as described previously [59]. A six-well plate was coated with 1.5 mL of 4% agarose (Gibco) in DMEM to form the bottom layer. The top layer consisted of 0.6% agarose containing 3500 cells/well. Once the top layer was solidified, it was covered with 2 mL media. After 3 weeks of incubation, 1 mg/mL 4-Nitro blue tetrazolium chloride (NBT) solution (Sigma-Aldrich) was added overnight and incubated at 37 °C to stain the colonies. The clones were assessed using Clono Counter software as described previously [60].

### 4.8. CD73 Activity Assay

The CD73 enzymatic activity of glioma cells was evaluated by the ability of the cells to convert AMP to ADO. GSCs were seeded at cell density of 1 × 10^5^ cells/mL and incubated with 10 μM AMP for 1 h at 37 °C. To evaluate the consequences of CD73 inhibitor APCP on the enzymatic activity 10 μM of APCP was added to the cells 10 min before adding AMP. Subsequently, cells were centrifuged and the amount of AMP and ADO in the supernatants was analysed by high-performance liquid chromatography (HPLC) (Shimadzu, LC-20AT; column: Nucleosil 1000-7 C18 250 mm). The absorbance was measured at 260 nm and 20 µl samples were injected while the column was kept at room temperature. Methanol and a 0.6 M K_2_HPO_4_/0.4 M KH_2_PO_4_ aqueous buffer (pH = 6) with a flow rate of 1 mL/min were used to separate the compounds [61]. The column was flushed with buffer for 30 min before each measurement. For the first 2 min, the mobile phase consisted of 100% buffer. Between 2 and 9.5 min, methanol was increasingly added up to a volume of 15.5%, which remained like this until min 17. Between minute 17 and 20, the buffer concentration increased linearly until reaching 100% and finally, the column was flushed with H_2_O for 5 min, adding up to a total run time of 25 min per measurement. Quantification of AMP and ADO values was done using a calibration curve of the standards.

### 4.9. In Silico Analysis of RNA Sequencing Data

RNA sequencing data were acquired from different anatomic structures of GBM from the Ivy Glioblastoma Atlas Project from the Allen Institute for Brain Science. Five tumour structures including the leading tumour edge, infiltrating zone, tumour parenchyma, hyperplastic blood vessels and microvascular proliferation were identified by H&E staining and compared to peri-necrotic zone and pseudopalisades. Fifty-five RNA samples were generated and used for sequencing. The data were retrieved in January 2018. Website: © 2015 (Internet). Available from: glioblastoma.alleninstitute.org.

### 4.10. Statistical Analyses

All data were obtained from three independent experiments and are the mean ± SD. The statistical significance was calculated using an unpaired student *t* test using the GraphPad Prism software version 8 (GraphPad Software, San Diego, CA, USA). Differences were considered significant for a *p* value of *p* < 0.05.

## Figures and Tables

**Table 1:** Primer sequences used in RT qPCR.

Name	Forward Primer (5′-3′)	Reverse Primer (3′-5′)
*β actin*	CCCAGCACAATGAAGATCAA	CGATCCACACGGAGTACTTG
*CD73*	TCTTCTAAACAGCAGCATTCC	CATTTCATCCGTGTGTCTCAG
*MMP2*	CCATCGAGACCATGCGGAAG	CCTGTATGTGATCTGGTTCTTG

## Supplementary Materials

The following are available online at https://www.mdpi.com/1424-8247/13/11/378/s1. Figure S1: Enzymatic conversion of AMP to ADO by CD73. Figure S2: HPLC analysis was used to determine AMP and ADO concentrations and thus the enzymatic activity of CD73. Figure S3: CD73 enzymatic activity does not affect GBMs survival. Figure S4: Pharmacological inhibition of the A_3_A receptor reduces GBM viability.
