# Supplementary material for: Enzymatic Activity of CD73 Modulates Invasion of Gliomas via Epithelial–Mesenchymal Transition-Like Reprogramming
Source: Pharmaceuticals (Basel). 2020 Nov 11;13(11):378. doi: 10.3390/ph13110378 (PMC7698190; doi:10.3390/ph13110378)
Supplement: Supplementary file 1 [file pharmaceuticals-13-00378-s001.pdf]

Supplementary materials:

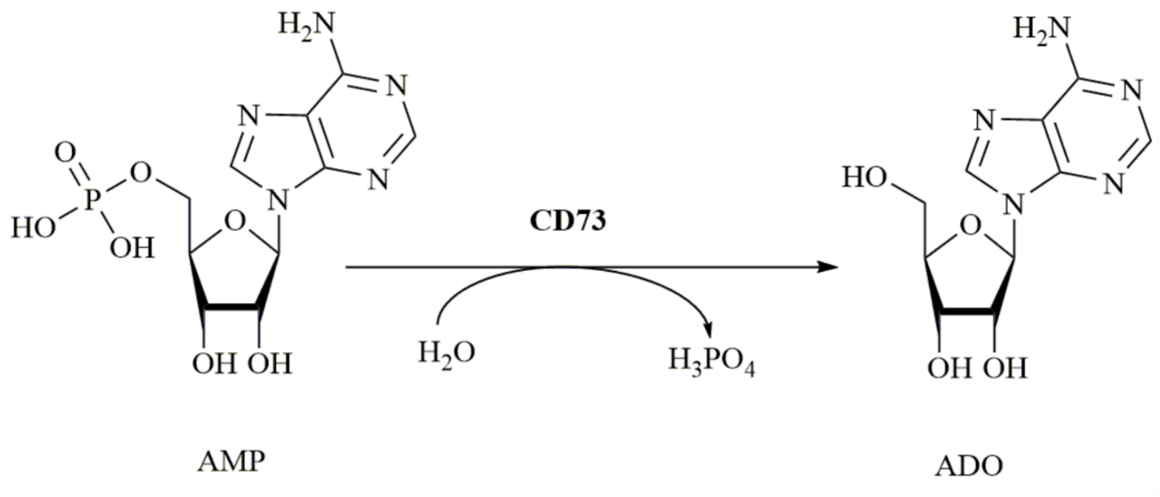

Figure S1. Enzymatic conversion of AMP to ADO by CD73.

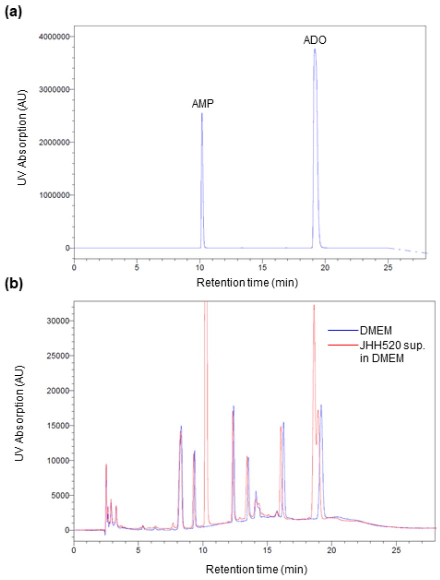

Figure S2. HPLC analysis was used to determine AMP and ADO concentrations and thus the enzymatic activity of CD73. Chromatograms of AMP and ADO standards (**a**) and of supernatant of JHH520 cells after incubation with AMP (**b**) are shown. After optimizing the separation conditions of our method, it was possible to identify the peaks corresponding to AMP (retention time: 10 min) and ADO (retention time: 19 min). Even though DMEM medium (b, in blue) presented further signals in the chromatogram, it was possible to locate AMP and ADO in the supernatant solutions (b, in red) and quantify their concentration through standard calibration.

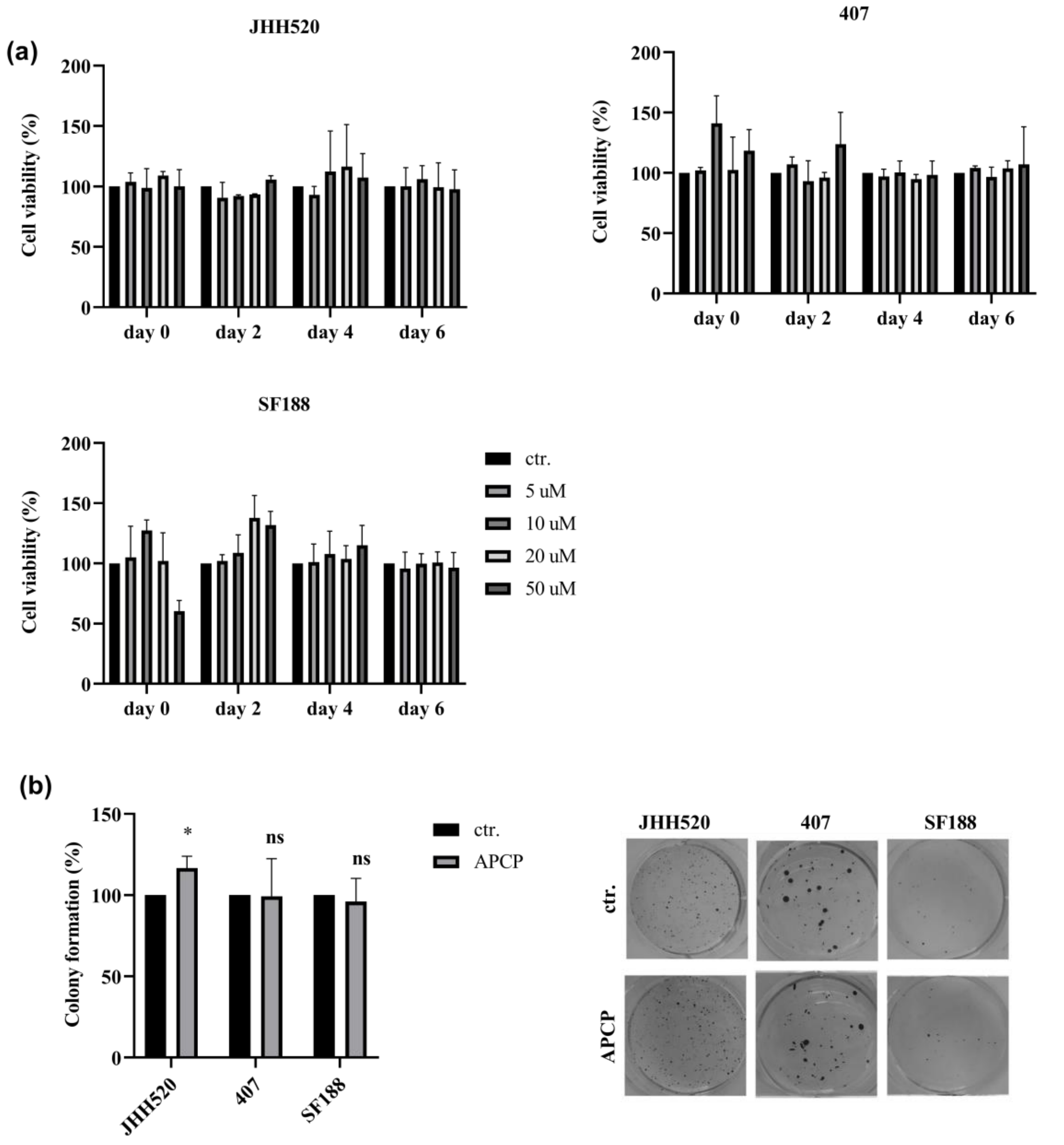

Figure S3. CD73 enzymatic activity does not affect GBMs survival. (**a**) Cell viability of GBMs upon treatment with APCP (1-50 μM) was not significantly decreased compared with untreated control cells (ctr.). (**b**) Inhibition of CD73 enzymatic activity with APCP (10 μM) did not affect the clonogenicity of JHH520, 407 and SF188 cells as assessed by soft agar assays. Results are the mean ±SD of three independent experiments performed in triplicate. *$p < 0.05$.*

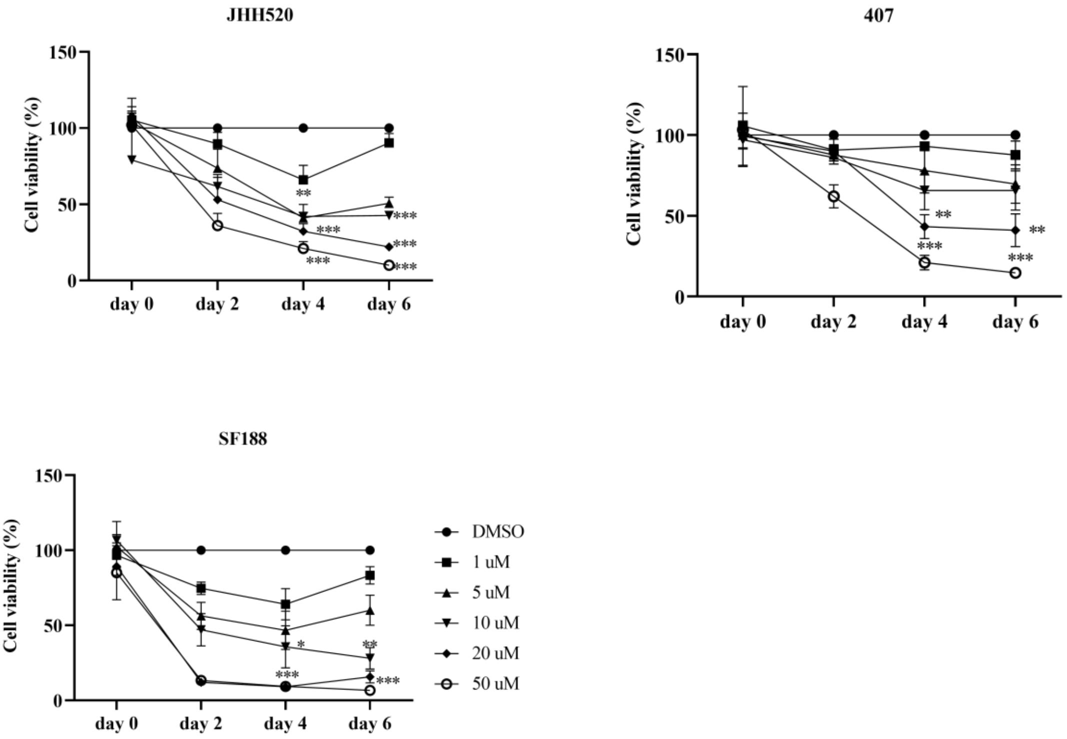

Figure S4. Pharmacological inhibition of the A_3A receptor reduces GBM viability. GBM cell lines (JHH520, 407 and SF188) were treated with increasing concentrations of MRS1220 (1-50 μM) and the cell viability was assessed using an MTT assay. Treatment with MRS1220 reduced cell viability in a dose-dependent manner ($p < 0.0001$). Results are the mean ±SD of three independent experiments performed in triplicate. Results are the mean ±SD of three independent experiments performed in triplicate. **p < 0.05, **p < 0.005, ***p < 0.0001.*
